# Low dose of flurbiprofen axetil decrease the rate of acute kidney injury after operation: a retrospective clinical data analysis of 9915 cases

**DOI:** 10.1186/s12882-020-1711-5

**Published:** 2020-02-14

**Authors:** Dong Wang, Shi-Kun Yang, Meng-Xi Zhao, Yong-Zhong Tang, Wen OU-Yang, Hao Zhang, Qin Liao

**Affiliations:** 1 Department of Anesthesiology, The Third Xiangya Hospital of Central South University, 138, Tongzipo Road, Changsha, Hunan China; 2 Department of Nephrology, The Third Xiangya Hospital of Central South University, Changsha, Hunan China

**Keywords:** Flurbiprofen, Acute kidney injury, Operation, Retrospective study

## Abstract

**Background:**

Flurbiprofen axetil (FA) is a commonly prescribed agent to relieve perioperative pain, but the relationship between FA and postoperative acute kidney injury (AKI) remains unclear. This study attempted to evaluate the effects of different dose of perioperative FA on postoperative AKI.

**Methods:**

A total of 9915 patients were enrolled for this retrospective study. The clinical characteristics and the prevalence of postoperative AKI among patients non-using, using low dose (50-100 mg), middle dose (100-250 mg) and large dose (≧250 mg) of FA were analyzed respectively. The impact of different dose of FA on postoperative AKI was analyzed using univariable and multivariate logistic regression analysis.

**Results:**

The prevalence of postoperative AKI was 6.7% in the overall subjects and 5.1% in 2446 cases who used FA. The incidence of AKI in low dose group was significantly less than that of non use group (4.5% vs 7.2%, *P* < 0.001), but the incidence of AKI in large dose group was significantly higher than that in the non-use group (18.8% vs 7.2%, *P* < 0.001). However, there was no significant difference between patients without using FA and subjects using middle dose of FA (7.2% vs 5.6%, *p* = 0.355). Multivariate logistic regression analysis showed that low dose of FA was a protective factor for postoperative AKI (OR = 0.75, *p* = 0.0188), and large dose of FA was a risk factor for postoperative AKI (OR = 4.8, *p* < 0.0001).

**Conclusions:**

The impact of FA on postoperative AKI was dose-dependent, using of low dose FA (50-100 mg) perioperatively may effectively reduce the incidence of postoperative AKI.

## Background

Acute kidney injury (AKI) is a syndrome with characteristic of the rapid loss of the renal function. AKI has a frequency of 1.9% in all hospital inpatients and is especially common in intensive-care unit of sepsis patients, in whom the prevalence of AKI is more than 40% [1]. Postoperative AKI is a common complication in various operation patients, and it occurs in approximately 6.3–7.4% of patients who undergo noncardiac surgery [2, 3]. It is associated with adverse outcomes including prolonged mechanical ventilation and high morbidity. Recently, numerous studies have investigated the pathogenesis for AKI after surgery, the present evidence indicates that the pathogenesis of postoperative AKI is mainly related to haemodynamic injury, systemic inflammation, renal hypoxia, ischemia-reperfusion injury and oxidative stress [4], and direct nephrotoxicity effects on the renal proximal tubule epithelial cells by various medication (e.g. iodinated contrast material, aminoglycoside antibiotics) is a vital cause for postoperative AKI, additionally, fluid depletion, renal neuroendocrine response to anesthesia and surgery itself are important reasons for postoperative AKI [5, 6].

Flurbiprofen axetil (FA) is an injectable non-selective cyclooxygenase (COX) inhibitor. It can selectively accumulate in surgical incision and inflammatory site to reduce the inflammation and pain because of composed emulsified lipid microspheres [7]. Perioperative intravenous administration of FA has been reported to be associated with a reduction of postoperative pain. Nonsteroidal anti-inflammatory drugs (NSAIDs) are known to affect renal function in susceptible patients by inhibiting the synthesis of vasodilating renal prostaglandins [8]. Previous studies have indicated that FA could ameliorate renal injury in the rats with 5/6 kidney ablation [9, 10], but some clinical data have showed that FA could induce renal papillary necrosis [11], and even acute tubulointerstitial nephritis [12]**.** However, a 52-week long time clinical study found that the effect of long-term FA application on renal function was small and subclinical [13]**.** Furthermore, the correlation between the effect of FA for perioperative analgesia on renal function and the range of dose safety is unclear. In order to explore the exact effects of FA on postoperative AKI, we performed this large retrospective study to guide future clinical applications of FA.

## Methods

### Participants and design of study

This was a retrospective clinical investigation of operation patients performed in the third Xiangya hospital of Central South University. We retrospectively reviewed medical records of all operation patients admitted from January 2012 to July 2017. The exclusion criteria were as follows: (1) subjects with age younger than 18 years. (2) subjects with any sepsis disease (e.g. urinary tract infections), tuberculosis. (3) subjects with acquired immune deficiency syndrome, or cancer. (4) all patients who underwent heart surgery or urologic surgery (including renal transplantion). (5) operation patients only using local anesthesia. (6) critically ill patients whose American Society of Anesthesiologists (ASA) grade was IV or V. (7) subjects with data missing of urine volume and serum creatinine (Scr). The ethics committee of the third Xiangya Hospital of Central South University has approved this study.

### Flurbiprofen axetil administration

The eligibility of the candidate was defined as those operation individuals who did not use any other NSAIDs except FA during the perioperative period. Patients receiving FA 48 h after surgery were excluded.

### Data collection

**Fig. 1:**
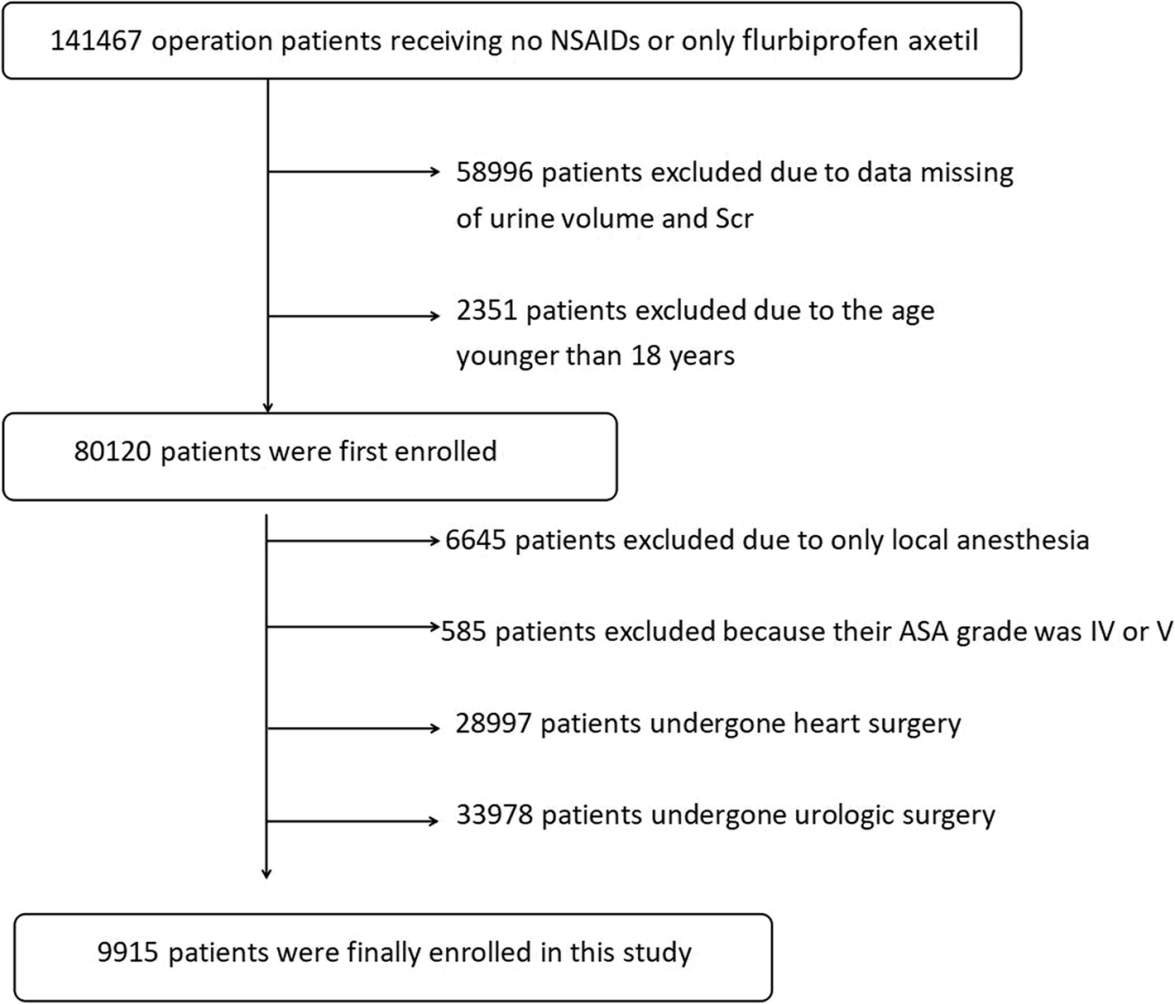
The data filtering process was shown in Fig. 1, we retrospectively reviewed medical records of all operation patients admitted from January 2012 to July 2017, there were 141,467 operation patients receiving flurbiprofen axetil, after significant filtering there remained 9915 patients finally enrolling in this study

The data filtering process was shown in Fig. 1. The information pertaining to demographics, past medical history, laboratory assessments, and hospital treatment has been reviewed. We searched medical records of all operation patients to collect the information of age, sex, preoperative hemoglobin (Hb) and Scr on pre-operation, ASA grade, surgery time, anesthesia method, surgery grade, emergency surgery, FA using, intraoperative haemorrhage, intraoperative erythrocyte transfusion, in fluids amount and out fluids amount, in addition, the personal history was included to analyse (e.g. history of diabetes, hypertension, preoperative medication about ACE inhibitors, angiotensin receptor blockers (ARB), calcium Channel Blockers (CCB) and diuretics.

### Definition

Postoperative AKI was diagnosed within 7 days after surgery based on changes in Scr, according to the Kidney Disease Improving Global Outcome criteria: a rise in Scr by ≥0.3 mg/dl within 48 h or an increase in Scr ≥1.5 times baseline within 7 days [14]. Scr before operation was defined as the lowest value obtained within 7 days prior to operation. KDIGO urine output criteria for AKI were not used since some patients received diuretic drugs during or after operation. We evaluated preoperative renal function by calculating the estimated glomerular filtration rate (eGFR). We used the four-variable Modification of Diet in Renal Disease (MDRD) formula to calculate eGFR = 175× Scr-^1.154^ × age^-0.203^ × 1.212 (if black) × 0.742 (if female). Preoperative chronic kidney disease (CKD) was defined as eGFR ≦90 ml/min/1.73m^2^. According to the National Nosocomial Infection Surveillance (NNIS) index, the surgery was divided into four grades (NNIS 0, NNIS1, NNIS 2, NNIS3) [15]**.** Different anesthesia techniques including general anesthesia and intraspinal anesthesia were used for these patients. According to American society of Anesthesiologists physical status classfication system, ASA grade was divided into grade I to grade V. Out fluids amount was defined as intraoperative blood loss and urine output. In fluids amount was defined as the intraoperative transfusion volume of blood and fluid. Net fluid input is defined as the subtraction between in fluids amount and out fluids amount. The dose of FA was the cumulative dosage from the beginning of anesthesia to 48 h after surgery.

### Statistical analysis

Statistical analyses were performed using the SPSS software, version 24.0 for windows program. The continuous results were expressed as mean values ± standard deviation ($\overline{x}$ ±SD), while the absolute and relative values (%) were presented for categorical dates. Data of baseline characteristics were analyzed using *t*-test or one-way ANOVA for continuous variables, while χ^2^ test and Fisher’s exact test was used for categorical variables. We used Least Significant Difference (LSD) t-test and Student-Newman-Keuls test for post-hoc analyses after one-way ANOVA compare to assess the differences of FA use among 4 groups. The logistic regression analysis was used to identify univariable and multivariable predictors for AKI. The adjusted relationship between FA and AKI was then modelled using multivariable logistic regression with adjustment for a priori selected risk factors for AKI. We reported adjusted odds ratios and associated 95% CIs and *p* values. All statistical tests were two-tailed, and *p* < 0.05 was considered significant.

## Results

### Comparison of clinical characteristics among patients with and without AKI

During the 6-years study period, 9915 operation patients were included in this study. Of these 9915 patients, 662 (6.7%) developed AKI as defined by the KDIGO criteria. In 662 AKI subjects and 9253 non AKI individuals, there were significant difference between the rate of preoperative hemoglobin, preoperative CKD, hypertension, diabetes, emergency surgery, surgery grade, intraoperative erythrocyte transfusion and intraoperative haemorrhage (all *p* < 0.001). In AKI group, the rate of patients who used ACE inhibitors, ARB, diuretics or CCB preoperatively were higher than those in non AKI group (*P* = 0.006, *P* < 0.001, *p* = 0.001, *p* < 0.001, respectively). In addition, there was a significant difference between AKI group and non-AKI group in the rate of male, age, general anesthesia and out fluids amount (*p* = 0.006, *p* = 0.048, *p* = 0.011, *p* = 0.037, respectively). While there was no difference about surgery time and in fluids amount in these two groups (*p* = 0.393, *p* = 0.769, respectively). The rate of ASA grade I, grade II and grade III in AKI group was similar compared with non AKI group (*p* = 0.122). There was no difference between AKI group and no AKI group about cumulative dose of FA and net net fluid input (*p* = 0.203, *p* = 0.156, respectively) (Table 1).

**Table 1:** Comparison of clinical characteristics among patients with and without AKI

Factors	Non AKI group (*n* = 9253)	AKI group (*n* = 662)	*P*-value
Fundamental state			
Male, n (%)	4299 (46.5)	271 (40.9)	0.006
Age (Year)	52.8 ± 14.5	53.9 ± 14.2	0.048
Preoperative hemoglobin (g/dL)	9.0 ± 5.2	8.1 ± 5.2	< 0.001
Hypertension, n(%)	1942 (21.0)	186 (28.1)	< 0.001
Diabetes, n (%)	580 (6.3)	70 (10.6)	< 0.001
pre-operative chronic kidney disease,n(%)	600 (6.5)	90 (13.6)	< 0.001
Preoperative serum creatinine, (μmol/L)	87.2 ± 113.0	92.2 ± 110.4	0.271
Preoperative medication			
ACE inhibitors, n (%)	438 (4.7)	47 (7.1)	0.006
ARB, n (%)	274 (3.0)	40 (6.0)	< 0.001
CCB, n (%)	2266 (24.5)	267 (40.3)	< 0.001
Diuretics, n (%)	158 (1.7)	23 (3.5)	0.001
Surgery-related factors			
In fluids amount (10 ml/24 h)	1142.1 (625.0–1542.7)	958.8 (625.0–1533.9)	0.769
Out fluids amount (10 ml/24 h)	333.3 (145.8–604.7)	312.5 (125.0–604.2)	0.037
Emergency, n(%)	1136 (12.3)	144 (21.8)	< 0.001
General anesthesia, n (%)	7684 (83.0)	575 (86.9)	0.011
ASA Grade:			0.122
I	666 (7.2)	50 (7.6)	
II	5949 (64.3)	400 (64.0)	
III	2638 (28.5)	212 (32.0)	
Surgery grade, n (%)			< 0.001
0	480 (5.2)	24 (3.6)	
1	3222 (34.8)	246 (37.2)	
2	5284 (57.1)	352 (53.2)	
3	267 (2.9)	40 (6.0)	
Surgery time, n (%)			0.393
< 2 h	3283 (35.5)	252 (38.1)	
2 h–5 h	5009 (54.1)	346 (52.3)	
> 5 h	961 (10.4)	64 (9.7)	
Intraoperative Erythrocyte transfusion, ml (%)			< 0.001
< 100	6825 (73.8)	426 (64.4)	
100–600	956 (10.3)	75 (11.3)	
601–1000	716 (7.7)	61 (9.2)	
> 1000	756 (8.2)	100 (15.1)	
Intraoperative haemorrhage, ml (%)			< 0.001
< 100	2578 (27.9)	224 (33.8)	
101–600	4905 (53.0)	296 (44.7)	
601–1000	818 (8.8)	51 (7.7%)	
> 1000	952 (10.3)	91 (13.7%)	
Cumulative dose of flurbiprofen axetil, (mg)	22.9 ± 47.5	20.5 ± 53.8	0.203
Net fluid input (10 ml/24 h)	706.6 ± 425.4	682.0 ± 450.0	0.156

### The relationship between different dose of flurbiprofen axetil and AKI

Based on the cumulative dose of FA from the beginning of anesthesia to 48 h after surgery, the patients was divided into four groups: non use group was defined as not using FA, low dose group was defined as 50 to 100 mg, the middle dose group was defined as 150 mg to 200 mg, while the large dose group was defined as no less than 250 mg. As shown in Table 2, the rate of AKI was 7.2% in non use group, in 2246 subjects using FA, the rate of AKI was 5.1%. In addition, the rate of AKI was significantly decreased to 4.5% in 1920 patients using FA at a dose of 50 to 100 mg, which was significantly less than that of non use group (*p* < 0.001). However, there was no significant difference between patients without using FA and subjects using FA at a dose of 150 mg or 200 mg (*p* = 0.355). On the contrary, in the subjects using FA at a dose of 250 mg or more, the rate of AKI was significantly increased to 18.8%, which was significantly higher than that in non use group (*p* < 0.001).

**Table 2:** The relationship between different dose of flurbiprofen axetil (FA) and AKI

Dose (mg)	Number (n)	Non AKI (n)	AKI (n)	Rate of AKI (%)	P*
All patients	9915	9253	662	6.7	
Non use	7469	6932	537	7.2	
Use of FA	2446	2321	125	5.1	
Low dose	1920	1833	87	4.5	< 0.001
Middle dose	462	436	26	5.6	0.355
Large dose	64	52	12	18.8	< 0.001

Note: P*: compared with the group of not using flurbiprofen axetil. AKI: acute kidney injury

### Comparison of clinical characteristics among patients using different dose of flurbiprofen axetil

As shown in Table 3, we found significant differences among patients using different dose of FA in the rate of male, age, hypertension, preoperative CKD (all *p* < 0.001), ASA grade (*p* = 0.001), general anesthesia (*p* = 0.006), preoperative medication of ACE inhibitor, CCB (all *p* < 0.001), ARB (*p* = 0.003), and diuretics (*p* < 0.001). In addition, we also found that the factors of emergency operation, in fluids amount, out fluids amount, surgery grade, surgery time, intraoperative erythrocyte transfusion, and intraoperative haemorrhage were different (*p* < 0.05). Finally, there was no difference in preoperative hemoglobin (*p* = 0.16) and the rate of diabetes (0.071) among the patients using different dose of FA.

**Table 3:** Comparison of clinical characteristics among the patients using different dose of flurbiprofen axetil

Factors	Non Use group (*n* = 7469)	Low dose group (*n* = 1920)	Middle dose group (*n* = 462)	Large dose group (*n* = 64)	P
Fundamental state					
Male, n (%)	3355 (44.9)	966 (50.3)	217 (47.0)	32 (50.0)	< 0.001
Age (Year)	53.6 ± 14.7	50.8 ± 13.4	50.8 ± 13.8	49.5 ± 15.7	< 0.001
Preoperative hemoglobin (g/dl)	8.9 ± 5.2	9.2 ± 5.3	9.0 ± 5.3	9.7 ± 5.6	0.16
Hypertension, n (%)	1728 (23.1)	319 (16.6)	75 (16.2)	9 (14.1)	< 0.001
Preoperative serum creatinine, (μmol/L)	91.7 ± 122.4	75.2 ± 74.8	74.2 ± 80.7	70.8 ± 43.1	< 0.001
Diabetes, n (%)	510 (6.8)	102 (5.3)	35 (7.6)	3 (4.7)	0.071
preoperative chronic kidney disease, n (%)	586 (7.8)	87 (4.5)	15 (3.2)	2 (3.1)	< 0.001
ASA Grade, n (%)					0.001
I	511 (6.8)	155 (8.1)	44 (9.5)	6 (9.4)	
II	4733 (63.4)	1285 (66.9)	288 (62.3)	43 (67.2)	
III	2225 (29.8)	480 (25.0)	130 (28.1)	15 (23.4)	
Preoperative medication					
ACE inhibitors, n (%)	407 (5.4)	62 (3.2)	13 (2.8)	3 (4.7)	< 0.001
ARB, n (%)	262 (3.5)	39 (2.0)	13 (2.8)	0 (0.0)	0.003
CCB, n (%)	2095 (28.0)	348 (18.1)	84 (18.2)	6 (9.4)	< 0.001
Diuretics, n (%)	160 (2.1)	17 (0.9)	4 (0.9)	0 (0.0)	< 0.001
Surgery-related factors					
In fluids amount	1041.7	1041.7	1333.3	1292.1	0.029
(10 ml/24 h)	(625.0–1520.8)	(666.7–1500.0)	(875.0–1750.0)	(875.0–1692.7)	
Out fluids amount	333.3	333.3	416.7	371.9	0.028
(10 ml/24 h)	(125.0–600.0)	(166.7–583.3)	(229.2–708.3)	(208.3–614.6)	
Emergency, n (%)	1065 (14.3)	169 (8.8)	41 (8.9)	5 (7.8)	< 0.001
General anesthesia, n (%)	6175 (82.7)	1623 (84.5)	408 (88.3)	53 (82.8)	0.006
Surgery grade, n (%)					< 0.001
0	425 (5.7)	72 (3.8)	6 (1.3)	1 (1.6)	
1	2581 (34.6)	744 (38.8)	126 (27.3)	17 (26.6)	
2	4211 (56.4)	1061 (55.3)	320 (69.3)	44 (68.8)	
3	252 (3.4)	43 (2.2)	10 (2.2)	2 (3.1)	
Surgery time, n (%)					< 0.001
<2 h	2845 (38.1)	595 (31.0)	79 (17.1)	16 (25.0)	
2 h–5 h	3870 (51.8)	1125 (58.6)	321 (69.5)	39 (60.9)	
>5 h	754 (10.1)	200 (10.4)	62 (13.4)	9 (14.1)	
Intraoperative erythrocyte transfusion, ml (%)					< 0.001
< 100	5409 (72.4)	1491 (77.7)	307 (66.5)	44 (68.8)	
100–600	782 (10.5)	183 (9.5)	56 (12.1)	10 (15.6)	
601–1000	603 (8.1)	123 (6.4)	45 (9.7)	6 (9.4)	
> 1000	675 (9.0)	124 (6.5)	54 (11.7)	4 (6.3)	
Intraoperative haemorrhage,ml (%)					< 0.001
< 100	2269 (30.4)	487 (25.4)	41 (8.9)	5 (7.8)	
100–600	3796 (50.8)	1056 (55.0)	307 (66.5)	42 (65.6)	
601–1000	635 (8.5)	187 (9.7)	41 (8.9)	6 (9.4)	
> 1000	769 (10.3)	190 (9.9)	73 (15.8)	11 (17.2)	

Note: *AKI* acute kidney injury, *Scr* serum creatinine, *ASA* American Society of Anesthesiologists

### Comparison of odds ratios of acute kidney injury across different dose group of flurbiprofen axetil by multivariate regression analysis

As shown in Table 4, multivariate regression analysis was performed to compare the odds ratio between the dose of FA and the incidence of acute kidney injury. After adjusting various interference factors based on three different models, we found that low dose of FA was protective factor for acute kidney injury (OR = 0.75, *p* = 0.0188), and large dose of FA was risk factor for acute kidney injury (OR = 4.8, *p* < 0.0001)。

**Table 4:** Comparison of odds ratios of AKI across different dose group of flurbiprofen axetil by multivariate regression analysis

Groups			
	Model 1 P	Model 2 P	Model 3 P
Non-use	1	1	1
Low dose group (50-100 mg)	0.61 (0.48, 0.77) < 0.0001	0.71 (0.56, 0.90) 0.0042	0.75 (0.59, 0.95) 0.0188
Middle dose group (150-200 mg)	0.80 (0.54, 1.19)0.2689	0.92 (0.61, 1.39) 0.6847	0.99 (0.65, 1.49) 0.9489
Large dose group (≥250 mg)	2.98 (1.58, 5.62) 0.0007	4.15 (2.18, 7.90) < 0.0001	4.80 (2.49, 9.26) < 0.0001

Model 1:Non-adjusted

Model 2:adjusted for age, sex, preoperative hemoglobin, hypertension, diabetes, use of ACE inhibitors, CCB and diuretics, general anesthesia, emergency, ASA grade, surgery grade, in fluids amount, out fluids amount. Hosmer-Lemeshougoodness test: *P* = 0.295

Model 3: adjusted for age, sex, preoperative hemoglobin, hypertension, diabetes, use of ACE inhibitors, ARB, CCB and diuretics, general anesthesia, emergency, ASA grade, surgery grade, in fluids amount, out fluids amount, surgery time, intraoperative erythrocyte transfusion, intraoperative haemorrhage and preoperative chronic kidney disease. Hosmer-Lemeshou goodness test: *P* = 0.123

Note: *AKI* acute kidney injury, *ASA* American Society of Anesthesiologists

### Compare of odds ratios of acute kidney injury across different dose group of flurbiprofen axetil by ordinal logistic regression analysis

According to the Kidney Disease Improving Global Outcome(KDIGO)criteria, AKI is divided into three grades. Grade 1: serum creatinine increased by ≥0.3 mg/dl within 48 h or increased 1.5–1.9 times baseline during 7 days. Grade 2: serum creatinine increased 2.0–2.9 times baseline. Grade 3: serum creatinine increased ≥3 times baseline or serum creatinine increased to 4.0 mg/dl (353.6 μmol/L) or initiation of renal replacement therapy. We divided the patients into three groups according to the stages of AKI:stage 0 = no AKI, stage1 = AKI grade 1, stage2 = AKI grade2 and 3. As shown in Table 5, ordinal logistic regression analysis was performed to compare the odds ratio between the dose of FA and the incidence of acute kidney injury. After adjusting various interference factors based on three different models, we found that low dose of FA was protective factor for acute kidney injury, and large dose of FA was risk factor for acute kidney injury.

**Table 5:** Compare of Odds Ratios of Acute Kidney Injury across different dose group of flurbiprofen axetil by ordinal logistic regression analysis

Groups	Model 1	Model 2	Model 3
	P	P	P
Non-use	0	0	0
Low dose group (50-100 mg)	− 0.0396 (− 0.0587,-0.0204) < 0.0001	−0.0117 (− 0.0215,-0.0019) 0.0198	−0.0194 (− 0.0385,-0.0003) 0.0463
Middle dose group (150-200 mg)	− 0.0262 (− 0.0621,-0.0096) 0.1513	−0.0025 (− 0.0208,-0.0158) 0.7908	−0.0064 (− 0.0419,0.0292) 0.7260
Large dose group (≥250 mg)	0.1458 (0.0520,0.2397) 0.0023	0.0991 (0.0504,0.1477) < 0.0001	0.1834 (0.0910,0.2758) 0.0001

Model 1: Non-adjusted

Model 2: adjusted for age, sex, preoperative hemoglobin, hypertension, diabetes, use of ACE inhibitors, CCB and diuretics, general anesthesia, emergency, ASA grade, surgery grade, in fluids amount, out fluids amount

Hosmer-Lemeshou goodness test: *P* = 0.279

Model 3: adjusted for age, sex, preoperative hemoglobin, hypertension, diabetes, use of ACE inhibitors, ARB, CCB and diuretics, general anesthesia, emergency, ASA grade, surgery grade, in fluids amount, out fluids amount, surgery time, intraoperative erythrocyte transfusion, intraoperative haemorrhage and preoperative chronic kidney disease

Hosmer-Lemeshou goodness test: *P* = 0.121

Note: AKI: acute kidney injury; We divided the patients into three groups according to the stages of AKI:stage 0 = no AKI, stage1 = AKI grade 1, stage2 = AKI grade2 and 3

*ASA* American Society of Anesthesiologists

## Discussion

It was reported that the rate of AKI was approximately 6.3–7.4% in patients who underwent noncardiac surgery [2, 3]. Similar to the above results, 662 patients (6.7%) developed AKI in our patients undergoing noncardiac surgery or nonurologic surgery.

The pathogenesis of postoperative AKI is complex and multifactorial. The use of nephrotoxic drugs was an important contributor for postoperative AKI, which has been demonstrated previously [6]. NSAIDs are the cornerstone of pain management in patients who have acute pain (eg, postoperative pain) for their analgesic and anti-inflammatory effects [16]. As a kind of targeted NSAIDs, intravenous administration of FA has been often used to reduce perioperative pain. Previous studies have explored the impact of FA on renal injury. However, the findings are controversial. Colome et al. found that FA could lead to renal papillary necrosis [11]. Similarly, Kaufhold et al. reported that a case of acute tubulointerstitial nephritis induced by FA [12]. Conversely, some animal experiments have indicated that FA could ameliorate renal injury in the rats with 5/6 kidney ablation [9, 10]. Further study identified that the impact of long-term FA application on renal injury was small and subclinical [13]**.** These findings indicated that the relationship between FA and kidney injury need to be further explored, and whether different dose of FA will generate beneficial or detrimental effects on AKI is unclear.

In this large single-center retrospective study, we explored the exact effects of FA on postoperative AKI, as a matter of fact, the subjects in the group of not using FA did not use any other NSAIDs for reducing postoperative pain, and for the first time we found that the impact of FA on postoperative AKI was dose-dependent, using of low dose of FA (50-100 mg) perioperatively was significantly associated with decreased incidence of postoperative AKI. This finding was opposed to the traditional concept: NSAIDs was an important cause of AKI both in children and adults [17, 18].

The mechanisms responsible for the different biological effect on renal function depending on different dose of FA is unclear. Large dose of FA may inhibit COX, interfering on arachidonic acid conversion into prostaglandins, reducing renal blood flow, causing tubular obstruction through crystal deposition, and inducing direct cytotoxicity or cell-mediated immune injury. It was reported that FA could induce renal papillary necrosis [11]**,** and acute tubulointerstitial nephritis [12]. Hence, large dose of NSAIDs have been implicated as causes of AKI, especially in the elderly. What we were more interested in was that low dose of FA perioperatively maybe an independent protective factor for postoperative AKI. It has been reported that increased renal expression of COX1 and COX2 in various nephropathies (e.g. lupus nephritis, heymann nephritis and renal ablation) [19–21]**.** COX derivatives may play an important role in the pathogenesis of progressive nephropathies. The potential role of prostanoids in the pathogenesis of progressive nephropathies has long been acknowledged. The stimulation of podocytes by complement fractions can increase the local synthesis of prostanoids. Similarly, non-immune mechanisms such as mesangial stretching can augment the expression of COX and enhance the production of its derivatives [22]. Accordingly, studies of the 5/6 renal ablation model showed increased urinary excretion of prostanoids per nephron [23]. It has been found that increased production of prostanoids can enhance inflammation and, therefore, accelerate renal injury. Goncalves et al. found that COX-2 could mediate inflammation and structural injury in the glomeruli [10]. In addition, part of the renal injury secondary to operation is a consequence of the inflammatory response with involvement of COX-2 and prostaglandins, the production and secretion of chemokines and cytokines (e.g. IL-1, IL-6 and TNF-α). So we speculated that low dose of FA could exert renal protective effects via reducing the inflammatory effects on the kidney [24, 25]. In addition, surgery and pain per se could result in activation of the renin-angiotensin system (RAS) and increased secretion of antidiuretic hormone, ultimately lead to hemodynamics abnormality [26, 27]. FA could alleviate pain, which maybe result in the restoration of the RAS and renal hemodynamics. What’s more, because of high affinity for inflammatory tissues, low dose of FA could accumulate mainly in surgical incision and inflammatory site and exert minor effect on the kidney. Further in vivo and in vitro study will be necessary to confirm this.

Because of a variety of reasons, there still exist several drawbacks in this study. First, our study enrolled all patients in a single center and thus external validity is limited. Second, AKI was determined according to the KDIGO criteria using the change in Scr in the present study, accurate urine output data at the general ward were not available. Although most of the previous studies did not use urine output criteria to diagnose AKI, the incidence of AKI might be changed if it is included. Third, we have excluded 58,996 patients who had missing data of Scr and urine volume, and the missing data might be from these patients who had low risk for developing AKI, therefore, this may reduce the accuracy of AKI incidence rate in the group of not using FA. Fourthly, the retrospective study design precludes the suggestion of any causal interference. Bias from unknown or unmeasured confounders may have influenced the results. In addition, due to a retrospective study and the large number of patients, it was difficult to collect the data such as the type of infusion liquid, vasopressors, contrast, proton pump inhibitor and intraoperative blood pressure, which may be the risk factors for AKI [28, 29]. The fifth, although we excluded cardiac and urologic surgery, we did not take the other type of surgery into account. All these can affect the use of FA and/or AKI. Finally, randomized controlled trials are needed to verify the effects of low-dose FA on AKI.

## Conclusion

In conclusion, our study showed for the first time that the impact of FA on postoperative AKI was dose-dependent, using of low dose FA (50-100 mg) perioperatively may effectively reduce the incidence of postoperative AKI. However, further animal experiment and multicentric large prospective studies will be needed to confirm this point.

## Data Availability

The data used to support the findings of this study are available from the first author and corresponding author upon reasonable request.

## Abbreviations

AKI: Acute kidney injury
ARB: Angiotensin receptor blockers
ASA: Anesthesiologists
CCB: Calcium Channel Blockers
CKD: Chronic kidney disease
COX: Cyclooxygenase
eGFR: Estimated glomerular filtration rate
FA: Flurbiprofen axetil;
Hb: Hemoglobin
KDIGO: Kidney Disease Improving Global Outcome
LSD: Least significant difference
MDRD: Modification of Diet in Renal Disease
NNIS: National nosocomial infection surveillance
NSAIDs: Nonsteroidal anti-inflammatory drugs
RAS: Renin-angiotensin system
Scr: Serum creatinine
